# Applying a community-based participatory research approach to improve access to healthcare for Eritrean asylum-seekers in Israel: a pilot study

**DOI:** 10.1186/s13584-017-0185-9

**Published:** 2017-11-15

**Authors:** Nora Gottlieb, Tomer Weinstein, Jonah Mink, Habtom M. Ghebrezghiabher, Zebib Sultan, Rachel Reichlin

**Affiliations:** 1 Technical University Berlin, Berlin, Germany; 2 MigrantHealth:IL, Tel Aviv-Yafo, Israel; 3 African Refugee Development Center, Tel Aviv-Yafo, Israel; 4 Eritrean Women’s Community Center, Tel Aviv-Yafo, Israel; 5 University of Illinois at Chicago, Chicago, Illinois USA; 6 Ben-Gurion University of the Negev, Beer Sheva, Israel

**Keywords:** Asylum seekers, Community-based participatory research, Healthcare access, Health insurance, Israel, Willingness to pay

## Abstract

**Background:**

Israel hosts approximately 50,000 asylum seekers, most of them from Eritrea. Exclusive policies restrict their access to healthcare. In 2013, local activists partnered with Eritrean asylum seekers to assess health needs as well as willingness to pay for health insurance among the Eritrean communities. This initiative was meant as a step towards jointly advocating access to Israel’s public healthcare system and towards strengthening collective efficacy among the asylum-seeking communities, as well as a first attempt to apply a community-based participatory research (CBPR) approach to migrant health research in Israel.

**Methods:**

Applying a CBPR approach, a 22-item survey was developed by a team of activists, academics and community members. It was administered by community members in four different cities. Cell weighting was applied to align sample estimates with the population distribution. The results were analyzed by means of a Generalized Linear Model. Six follow-up interviews and two focus group discussions helped interpret the quantitative findings and provided additional information.

**Results:**

The results from 445 questionnaires show that most (95%) asylum seekers are interested in purchasing health insurance. To this end, more than half of the respondents are willing to invest up to 300 NIS monthly, which represents a significant part (5–7.5%) of their median monthly income. Willingness to pay for health coverage was independent from employment and income; it was significantly associated with city of residence – which translates into current healthcare options - understanding of health insurance, and health seeking motives. The study further highlights the role of labor policies in shaping not only asylum seekers’ access to care but also psychosocial wellbeing.

**Conclusions:**

The study provides initial evidence for asylum seekers’ willingness to pay monthly contributions into a public health insurance scheme, in spite of economic hardship, and it points to understanding of and trust in the healthcare system as a central factor influencing willingness to pay. The outcomes of this initiative further offer some support for the potential of CBPR to enhance research into the health of marginalized populations and, moreover, to counter their social exclusion through capacity building and strengthening of collective efficacy.

**Electronic supplementary material:**

The online version of this article (10.1186/s13584-017-0185-9) contains supplementary material, which is available to authorized users.

## Background

The number of forced migrants is at its highest level since WWII [1]. Demands to address the needs of displaced persons arise out of ethical urgency as well as pragmatic concerns. Delays in addressing asylum seekers’ healthcare needs can cause avertable suffering as well as increase social and financial costs [2, 3]. Community-based participatory research (CBPR) can be a powerful way to assess asylum seekers’ health needs while, at the same time, acknowledging, engaging and building capacity within this population. This paper describes the results of a CBPR project with Eritrean asylum-seeking communities in Israel so as to provide a tangible example of confronting the global challenge of promoting migrants’ health.

Israel is one of the countries with the highest rate of immigrants per population worldwide [4]. However, it is essential to distinguish between the different groups of (im)migrants as there are marked differences as regards legal status and rights. Immigrants of Jewish descent, upon their arrival in Israel, enjoy immediate citizenship status and the full array of related rights, including inclusion in the National Health Insurance (NHI) scheme. For other, non-Jewish migrants there is barely any pathway to permanent legal status [5] and, with permanent residence status being the eligibility criteria for NHI membership, they are excluded from health rights. Alongside asylum seekers, the latter group comprises mainly authorized and unauthorized labor migrants. The law obliges employers of all non-citizen workers, whether authorized or not, to purchase private for–profit health coverage for their workers to ensure access to healthcare services [6]. This insurance scheme was put in place for labor migrants; yet today it also applies to asylum seekers. However, as for other low-wage minority worker populations across the globe, the formally held health rights are often not actualized [6–8].

It is difficult to know the exact number of asylum seekers in Israel, also due to the use of different definitions and terminologies, including the widespread term “infiltrators” [9]. For the purpose of this paper, we will apply the term “asylum seeker” to persons who have entered Israel from another country and have requested state sanctuary and/or are non-deportable and therefore fall under the state’s “temporary group protection”. Approximately 54,000 asylum seekers lived in Israel in 2013. Out of these, an estimated 65% (35,000) were from Eritrea. The “temporary group protection” status granted to them by the Israeli Ministry of Interior defers deportation; however it does not endow them neither with work permits nor access to healthcare [10, 11].

Certain public healthcare services such as basic primary maternal and child health services and the diagnosis and treatment of sexually transmitted infections and tuberculosis are available universally and free of charge [12–14]. Emergency care is provided unconditionally by public hospitals; yet uninsured patients are subsequently billed. As regards affordable primary care, many asylum seekers rely on the services offered by two charitable walk-in clinics in South Tel Aviv-Yafo. One is the NGO-run “Open Clinic”, which serves uninsured populations since 1998. The other “Refugee Clinic” was opened in 2009 by the Israeli Medical Association with support from the Israeli Ministry of Health. At both clinics a limited range of services is available at low rates or free of charge. Beyond that, Palestinian medical facilities in East Jerusalem offer comparably inexpensive rates [14, 15]; yet accessing them can be problematic in terms of time and travel costs. The few existing studies on the health of asylum seekers in Israel point to considerable delays in treatment onset [12, 14] and substantial unmet reproductive and mental healthcare needs [16, 17]. A special report by the State Comptroller [10] notes that “without adequate health care in the community the medical needs of some of the non-deportable foreigners are neglected until they become matters of emergency care.” (p.63) It further points out that “[t] he absence of [corresponding] budgetary resources is liable to harm also the hospitals’ economic stability” (ibid.) and calls on the Israeli government to offer a comprehensive solution “as soon as possible” (p.67).

The Israeli National Health Insurance Law includes a clause (§56 a(1)d) authorizing the Minister of Health to “determine special arrangements” for the expansion of health entitlements to non-resident populations [18]. Exceptional semi-public insurance arrangements have been made for non-resident children and, in the city of Eilat, for hotel workers. In the former case, parents can insure their children directly with one of the public Sick Funds, which, against payment of a state-subsidized monthly premium, provides access to services akin to NHI. Similarly, in Eilat - a city that traditionally holds a special status in terms of tax and work arrangements due to its geographic location and tourism industry – hotels can insure non-resident workers directly with a Sick Fund [19]. This benefit is included in the worker’s paystub. Based on these two precedents, the initiative described here aims to achieve a similar group-based arrangement for the general asylum-seeking population in Israel.

Community-based participatory research (CBPR) is based on the conceptual foundations of social justice, self-determination and empowerment [20]. The approach has been shown to engage marginalized communities and to effectively translate this engagement into practice by “placing political participation as the bridge between evidence and policy” [21]. Our project incorporated key tenets of CBPR by addressing the Eritrean asylum-seeking communities’ expressed desire to improve access to primary healthcare, by jointly working towards a solution, and by using the CBPR approach itself to tackle a structural cause for health inequity, namely exclusion from the political discourse. Given this framework, desired outcomes at the community level include the collective ability to mobilize resources to assert needs and improve wellbeing [20, 21].

In the summer of 2013 the asylum-seeking communities in Israel were demonstrating growing collective efficacy as they organized for political change. Among other demands, Eritrean community leaders underscored the need for access to primary care. Local activists who were already engaged with the Eritrean communities were working on ways to address the issue together. The goals of the initiative described here were: 1) to generate data that can inform migrant health policies in Israel and enhance advocacy efforts for greater inclusion; 2) to support the asylum-seeking communities’ collective efficacy and social inclusion; and 3) to simultaneously test the feasibility and added value of CBPR in the given context. The following sections will describe the CBPR approach to developing the research tool, as well as findings from the survey and follow-up interviews.

## Methods

The team consisted of two local activists and nascent social entrepreneurs, a public health nurse and academic, and four Eritrean community partners, all of them fluent in English and/or Hebrew. At the time the nurse/academic became involved, the survey was already in development. Importantly, there was no intent for this inquiry to be a rigorous academic pursuit. However, given the nurse’s/academic’s experience in CBPR, the team felt that this would be a helpful orientation to guide this initiative and possibly increase relevance and reach. During initial team meetings personal perspectives and narratives were exchanged. This process provided important context to the study and, at the same time, fostered trust and a sense of collective efficacy as the team realized possibilities that could evolve from synergizing each partner’s educational and social wealth.

Over a five-week period the team designed a survey tool that included open and closed questions addressing health needs and access to care, as well as interest and ability to invest in health insurance. The community partners added constructs that they deemed relevant such as working conditions and experiences of discrimination and stress; they also ensured that items were worded in a culturally appropriate way. The final instrument consisted of 22 items with an additional four questions for families with children (see Additional file 1). The community partners translated the survey from English into Tigrinya and then recruited Eritrean community members to administer the survey.

As a next step, the surveyors were trained in two three-hour sessions. Training sessions were executed with an emphasis on reciprocal learning while providing background on the project’s purpose, basic knowledge on the healthcare system, research ethics and survey administration techniques. The sessions offered an opportunity to further understand the Eritrean communities’ views on health, as well as their health-seeking behaviors, which helped finalize the last iteration of the survey. Given that the concept of health insurance may not be commonly understood among this population, the survey screened participants among other things for their understanding of health insurance as well as their willingness to pay, on a monthly basis, for a package of healthcare services – including access to primary care, specialty care, acute hospital care and pharmaceutical benefits. Principal health concerns were summarized into three main categories with the option to write-in an alternative. The categories accounted for stress and anxiety, chronic health conditions, and acute illness or accidents (see question 21, Additional file 1). Participants were asked to self-report and select the category that represented the primary health concern for which they usually seek care.

In total, there were four surveyors from Tel Aviv-Yafo, two from Ashdod, two from Jerusalem and two from Eilat. These four cities were included in the survey for representing the largest Eritrean communities in Israel, as indicated by official data [11] as well as the community partners’ tacit knowledge. All surveyors were male. They received compensation for taking part in training sessions as well as a modest compensation for implementing surveys (200 NIS per 100 questionnaires). The surveyors administered the questionnaire in their respective communities in July 2013. Each surveyor was asked to approach 100 Eritrean asylum seekers above the age of 17 years.

**Fig. 1:**
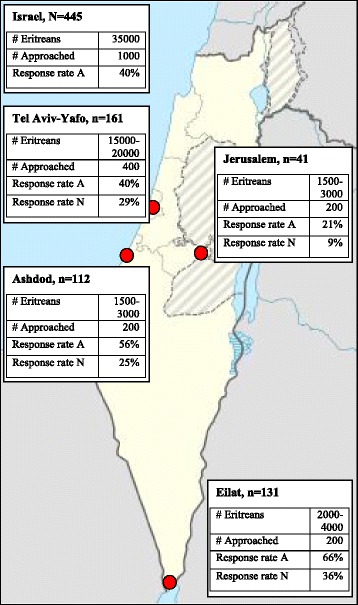
Map of Israel with sampled cities and response rates. # Eritreans: estimated number of Eritrean asylum seekers living in the respective location. # Approached: number of potential survey respondents (Eritrean asylum seekers >18 years) who were approached by the surveyors. Response rate A: as calculated from the number of respondents (N, n)/# Approached. Response rate N: as calculated from the number of respondents from one city (n)/total number of respondents (N)

The data was coded in EXCEL and analyzed using R 3.2.3 software [22]. The above described sampling strategy resulted in a relative undercoverage of the Eritrean asylum-seeking population in Tel Aviv-Yafo (representing approximately 60% of the Eritrean asylum-seeking population in Israel) and a relative overcoverage of the populations in Ashdod, Eilat and Jerusalem (representing approximately 10% of the Eritrean asylum-seeking population in Israel each, see Fig. 1). We therefore applied cell weighting to compensate for under-/overcoverage and make the statistical estimates represent the population of inference as closely as possible [23]. The following results section presents the weighted estimates; Table 1 provides the latter estimates alongside the unweighted survey results. For bivariate analysis we used Fisher’s exact test with simulated *p*-value based on 2000 replicates. All statistical tests were two tailed and considered significant when *P* < 0.05.

**Table 1:** Survey respondents’ characteristics and weighted population estimates

Characteristic	Survey response (*N* = 445)	Weighted estimate
Ratio of people aged under 35 years	87% (384)	85%
Ratio of men	78% (342)	79%
Average length of stay in Israel	3.43 (±1.33) years	3.36 (±2.75) years
Usual reason for seeking healthcare:		
Acute health problems	44% (172)	57%
Chronic health problem	20% (78)	11%
Stress, constant worry, trouble sleeping	34% (131)	31%
Employment type:		
Day labor/Contingent jobs	12% (51)	16%
Full time without paystub	32% (143)	37%
Full time with paystub	53% (233)	43%
Average monthly income:		
≤ 4000 NIS	40% (171)	38%
4001–6000 NIS	53% (226)	55%
> 6000 NIS	7% (31)	7%
Willingness to pay for health insurance:		
≤ 100 NIS	39% (173)	40%
101–300 NIS	56% (248)	54%
> 300 NIS	2% (7)	1%
Not willing to pay for health insurance	3% (14)	5%
Auxiliary services deemed important/very important		
Medical interpretation	96% (423)	96%
Instructions on medical regimens	97% (425)	97%
Information on health rights	97% (427)	98%

To further examine the respondents’ willingness to pay for health insurance (WTP), we designed a Generalized Linear Model with binomial error structure and logit link function [24]. As the response variable we set the amount that respondents named as the “fair price” they would pay for health insurance (≤100 NIS, >100 NIS). Fixed effects included types of employment (contingent jobs/day labor, full-time without paystub, full-time with paystub), income levels (<2000 NIS, 2001–4000 NIS, 4001–6000 NIS, >6000 NIS), and levels of understanding of health insurance (1 = “not at all clear” to 5 = “very clear”). We controlled for random effects of the following variables: age group (18–24 years, 25–35 years, >35 years), gender, city of residence (Ashdod, Jerusalem, Eilat, Tel Aviv-Yafo), length of stay in Israel (in years), and main reasons for seeking care (categorized as “acute illness”, “chronic illness”, “stress/constant worry/trouble sleeping”, and “other”). The full model was compared to its corresponding null model (containing only the intercept and random effects) using a likelihood ratio test [25, 26]. To examine the effect of each tested variable on WTP separately we excluded fixed and random variables, one at a time, from the dataset and compared the reduced model’s deviance with that of the full model.

Six follow-up interviews and two focus group discussions with a convenience sample of Eritrean community members helped interpret the survey findings. Individual and group interviews were documented through extensive note-taking. The texts were coded manually, applying memoing and open coding strategies during first readings and subsequently, with the progress of the analysis, more focused coding [27].

## Results

A total of 445 surveys were completed: 161 (36%) in Eilat, 131 (29%) in Tel Aviv-Yafo, 112 (25%) in Ashdod and 41 (9%) in Jerusalem. Assuming that 200 people were approached in each city, response rates were highest in Eilat (66%) (see Fig. 1). According to our weighted estimates, four out of five Eritrean asylum seekers are male and 85% are under 35 years old. More than half of the Eritrean asylum seekers said that usually acute health issues were their health concern and reason to seek care; while only one in ten reported a chronic health problem. Almost a third of the Eritrean asylum seekers said that they experience stress, constant worry and/or trouble sleeping. Survey responses and weighted estimates are outlined in Table 1.

The results show that, at the time of the survey, most asylum seekers were working. Fourty-three percent were in formal full-time employment (working full-time and receiving a monthly paystub); 37% were in informal full-time employment (no paystub); and 16% earned their living by contingent day-labor jobs. More than half had an average monthly income of 4,001 to 6,000 NIS; 38% earned 4,000 NIS or less per month.

According to our survey, the clear majority (95%) of Eritrean asylum seekers there are interested in joining a health insurance scheme. More than half considered a “fair” monthly contribution for an insurance equivalent to NHI to lie between 100 and 300 NIS. Another 40% were willing to pay up to 100 NIS. Only 5 % were unwilling and/or unable to pay for health insurance.

For our model (investigating the factors that influence WTP), all respondents with missing values in the WTP categories were excluded. This lead to a sample size of 299. Its deviance from the null model reached marginal significance (likelihood ratio test: χ2 = 9.2, df = 4, *P* = 0.056). The model found neither employment type nor income level to significantly shape WTP (see Table 2). The factors that showed clear effects were better understanding of health insurance (*P* = 0.035) and city of residence (*P* < 0.001), with respondents from Ashdod and Jerusalem being significantly more willing to invest in health insurance (see Table 2). Also respondents’ usual care seeking motive emerged as a significant determinant of WTP (*P* = 0.018): Respondents who reported seeking care mostly for acute health problems were willing to pay more for health insurance as compared to respondents reporting chronic and mental health problems (see Table 2).

**Table 2:** Generalized Linear Model with binomial error structure and logit link function investigating the factors that influence Eritrean asylum seekers’ willingness to pay for health insurance

Fixed effect	Effect ± SE	LRT- χ2	df	*P*
**Employment type**		4.563	2	0.102
Day labor	0 ± 0			
Fulltime without paystub	0.789 ± 0.560			
Fulltime with paystub	1.116 ± 0.553			
**Income level**	0.089 ± 0.278	0.101	1	0.751
**Level of understanding of health insurance**	0.225 ± 0.108	4.434	1	**0.035**
**City of residence**		44.728	3	**<0.001**
Ashdod	0 ± 0			
Jerusalem	-0.453 ± 0.845			
Tel Aviv-Yafo	-1.789 ± 0.468			
Eilat	-2.543 ± 0.480			
**Usual reason to seek healthcare**		10.050	3	**0.018**
Acute illness	0 ± 0			
Chronic illness	-0.155 ± 0.418			
Stress, constant worry, trouble sleeping	-0.583 ± 0.364			
Other	-1.377 ± 0.481			
**Length of stay in Israel**	0.186 ± 0.122	2.330	1	0.127
**Age group**	0.292 ± 0.251	1.367	1	0.242
**Gender (male)**	0.316 ± 0.362	0.763	1	0.383
**Intercept**	-1.087 ± 0.951			

Significant *p*-values are in bold

Our results show that most asylum seekers would value information and navigation services such as interpretation, instructions concerning treatment regimens, and information about health rights (rated as important or very important by 96, 97 and 98% respectively). In the follow-up interviews, participants underscored that *“[o]verall, [Eritrean] people have little knowledge about health insurance. In Eritrea, there is now car insurance. This is what people know in terms of insurance.”* They therefore confirmed the role of the said services, saying that *“people want to know what their rights are.*” They also made clear that

*“[i]t is very important to also give education to them [healthcare providers]. For example, in the health center in [South Tel Aviv], the person at the reception can tell you, ‘I am not willing to see a refugee.’ It happened to me twice… They have to be educated: If you’re paying, you deserve it. You have rights... They have to give us services like [to] everybody [else].”*

Disrespectful treatment from health service providers was a recurrent theme throughout the follow-up interviews and participants described it as a major deterrent to access mainstream healthcare. They explained that *“there are many people who are willing to buy insurance; and many already have it and pay regularly. But there is this discrimination... For these reasons they are discouraged to go.”* Participants further reported on problems with the current private for-profit labor migrant health plans. For example, they criticized how insurance companies take advantage of legal loopholes to evade covering more substantial health costs: *“[I]f people need continuous treatment or are in the hospital, they [insurance companies] terminate the contract, even if people want to continue to pay.”* Such statements exemplify how negative experiences diminish trust in Israeli healthcare providers among the asylum-seeking communities. Participants warned that access to insurance alone will not guarantee buy-in. They explained that a new insurance arrangement would have to correct systemic shortcomings and reliably offer quality healthcare in order to be successful. Eventually, many participants summed up their expectations of health insurance by underscoring that *“[t]he key is really the [providers], the service. If we pay equal money like Israelis, we want equal services.”*

Finally, our results provide some indication that the exceptional migrant labor and health policies in the city of Eilat have positive implications not only for asylum seekers’ access to care, but also more broadly for their wellbeing: They show the uneven distribution of formal full-time employment between the four cities, with 73% (116) of respondents from Eilat having a formal full-time job as compared to 14% (51) of respondents from Ashdod, 44% (18) from Jerusalem and 37% (48) from Tel Aviv-Yafo respectively (Fisher’s exact test, *n* = 441, *P* < 0.001). Formal employment, in turn, leads to employment-based health insurance and, in the case of Eilat, allows integration into the public healthcare system. Correspondingly, our findings show that a clear majority of respondents from Eilat (98%, 155) seek care from a local Sick Fund, i.e., through the public healthcare system; whereas in other cities, respondents named as their main healthcare providers charitable clinics (e.g. 45% (53) of respondents from Tel Aviv-Yafo) or East Jerusalemite providers (e.g. 60% (24) of respondents from Jerusalem). Among the respondents from Ashdod 28% (31) reported foregoing care in case of need (as compared to 1% (1) in Eilat, 0% in Jerusalem, and 2% (2) in Tel Aviv-Yafo). Moreover, formal employment was shown to mitigate some psychosocial stressors: While almost half of the respondents (48%, 199) described being worried or very worried about losing their job if they had to request a sick leave, these fears significantly decreased with formal employment (Fisher’s exact test, *n* = 423, *P* = 0.002).

The research team continues to work on asylum seekers’ access to care, yet systemic change is still forthcoming. Since this assessment, subsequent encounters with the community partners and surveyors demonstrated great enthusiasm, commitment and continued engagement. During follow-up interviews, they expressed deep satisfaction with the study’s approach, noting that “*finally someone relates to us as partners and potential [insurance] customers and not as hapless victims!*” Statements like this illustrate the empowering effects of the CBPR approach and its potential to build a more sustainable foundation for action than traditional research approaches, through the communities’ engagement and ownership of the initiative.

## Discussion

The results of this study support that a CBPR approach in a marginalized community can be effective in producing concrete information with a broad reach and in culturally appropriate ways, thus strengthening scientific evidence [28]. The fact that 445 survey responses from asylum seekers in four different cities could be collected is a success in itself, especially in light of the communities’ daily hardships. The data illustrates an average young age and a large ratio of men - characteristics that usually render a population attractive for insurance agencies as they translate to low risk paying members. In line with the “healthy migrant effect” hypothesis [29], previous research [12, 30] has reported that this is a generally healthy population. However, as our study shows, one should also note substantial prevalence of symptoms potentially associated with mental health needs. These may be related to experiences before, during and after migration [16, 17]. With regard to post-migration risk factors, our findings support prior studies [14] showing that precarious employment and the related social insecurity are major stressors in the lives of many asylum seekers, compounding the threats related to living in legal instability and a largely hostile environment. It is thanks to the community partners’ insertion that our study was able to capture initial information on the role of employment structures and working conditions. This is an area that deserves greater attention in migrant health research [31].

Most importantly, the survey results demonstrate that a clear majority of Eritrean asylum seekers in Israel are interested in purchasing health insurance, as illustrated by their willingness to pay up to 300 NIS per month, an amount that corresponds to 5–7.5% of the median monthly income in these communities. By way of comparison, the current health tax rate for Israeli citizens with a comparable income is 3.1–5% [32]. The private labor migrant healthcare scheme offers a slightly limited scope of services compared to NHI for rates as low as 120 NIS monthly [19]. In many migration-receiving countries, a dominant argument in the immigration debate is migrants’ alleged goal of exploiting the host country’s social system [33, 34]. To our knowledge, few studies have addressed the above concern with quantifiable data from the migrants’ perspective. Gonzáles Block et al. and Bustamante et al. examined willingness to pay for a binational insurance scheme among Mexican immigrants in the US [35, 36]. Their research demonstrates, much in line with similar studies in low-income, informally employed and/or rural populations [37–39], that considerable segments of the studied populations are interested in buying into insurance schemes. They found income levels and employment status to be central predictors of WTP. The perceived benefits of an insurance scheme such as accessibility and quality of services were shown to be another important consideration in the decision whether or not to join a healthcare plan. None of the above cited studies reported any evidence for moral hazard. And while our finding on chronically ill respondents’ *lesser* willingness to purchase health insurance is puzzling and cannot be explained here, it also clearly contradicts the moral hazard argument. In our study, those respondents who usually seek care for acute medical issues appeared *more* willing to pay for health insurance as compared to those with a chronic disease. This may be related to the latter’ negative healthcare experiences in Israel or to their socioeconomic situation. Yet, our study does not offer a well-founded explanation; this aspect warrants more targeted investigation.

The role of employment and income as key determinants of WTP is not supported by our study results. Instead they indicate that, in the given context, some migrants face such difficulties in accessing care that they are willing to pay for health insurance in spite of economic instability and privation. This seems to be the case particularly for asylum seekers living in Ashdod (where no primary healthcare options for asylum seekers are in place) and in Jerusalem (where most asylum seekers resort to East Jerusalemite medical facilities). Our study results do, however, confirm the important role of migrants’ understanding of the concept of health insurance, as well as acceptability and quality of care, namely (perceived/expected) discrimination from health staff. In the particular case described here, any new healthcare scheme will first have to (re-)build positive relationships between the asylum-seeking communities and healthcare providers. Initiatives that commence with community engagement like the one described here may be a good start.

In light of our study’s findings, the labor and health policies towards asylum seekers in Eilat might serve as a role model for the rest of the country. Our results indicate that more stable employment positively interacts with psychosocial health; plus, thanks to Eilat’s employment-based semi-public insurance scheme, they imply consistent access to healthcare. At the same time asylum seekers in other cities were shown to resort to a mix of alternative solutions or to forego healthcare altogether - factors that have been shown to ultimately increase health costs [2, 3]. Moreover, our study indicates that the opportunity to gain an understanding of and trust in public healthcare provision is key to asylum seekers’ readiness to invest in health insurance. In sum, our study helps to show that most migrants, rather than seeking hand-outs, want a “fair” insurance scheme that will allow them to contribute their share and, in return, enjoy the benefits of adequate, equitable healthcare provision.

## Conclusions

With an ear to the ground and the ability to leverage its members’ educational, social and cultural wealth, this first attempt of a CBPR initiative with asylum seekers in Israel was able to capture initial evidence that can be used to negotiate a migrant insurance plan. One of the goals of the initiative was to empirically examine whether a majority of asylum seekers would invest in a health insurance scheme, if they were given the opportunity. The initiative has accomplished this goal and its findings can be used to buttress advocacy for asylum seekers’ inclusion. That process is still underway. Facilitating access to healthcare for non-Jewish migrants in Israel is especially complex given the unwavering covenant to the state’s Jewish identity. Ethno-national concepts of citizenship and rights frame current policies of in-/exclusion, distinguishing between those who deserve membership and protection by the welfare state and undeserving others [6, 19, 40]. Another goal of the initiative was to test the feasibility of CBPR with a marginalized migrant community in the Israeli context and to demonstrate the added value of this approach. Given its initial outputs and successes, the initiative could serve as a basis for more rigorous and extensive follow-up studies. Finally, our study intended to support the Eritrean communities in more indirect ways as well, through leveraging collective efficacy and supporting participation in the political discourse. We hope that it has thus made a modest contribution to the asylum-seeking communities’ strife for civil and social rights, as exemplified by grassroots organizing efforts, including country-wide strikes and mass-demonstrations, that took place in the following months and received sympathetic media coverage and widespread support from Israeli employers [41, 42]. Future CBPR efforts may prove to be beneficial on several levels, including 1) generating datasets that will allow for more in-depth analysis; 2) contributing to grassroot organizing; and 3) strengthening advocacy for the social inclusion of marginalized communities. Lastly, developing a workforce prepared to practice CBPR within communities, NGOs, businesses, as well as within the academic world will be key to sustaining and formalizing this approach.

Several limitations underlie this study with regard to sampling strategies and tools to measure community-level outcomes. Community partners were recruited through convenience and snowball sampling with few starting points. For feasibility reasons, only Hebrew and/or English speakers were included in the research team. Moreover, all surveyors were male as the research team’s efforts to recruit female community members remained unsuccessful. The main reason for this failure is probably that most Eritrean women, bearing the triple burden of income generation, reproductive work and community functions, cannot accept yet another responsibility. The above described factors may have led to selection bias; i.e. the study may not capture the full range of characteristics and perspectives within the Eritrean communities in Israel, especially in light of their internal fragmentations. Moreover, those who were more concerned with medical care in first place may have been more inclined to participate in the survey. As a result, our study may overestimate the burden of illness in the study population.

Strongly skewed outcome distributions in the survey results impeded the statistical analysis. In a follow-up study, small changes in the survey instrument could easily resolve this shortcoming. Our results on long-term outcomes are limited mainly because the original initiative was focused on its needs assessment component. It would have been beneficial to evaluate the participants’ experience and long-term benefits more systematically through thorough qualitative data collection at the beginning, the end, and 6 months to a year after the study. The field of evaluating CBPR and the participatory process is newly evolving. As the respective tools [43] are tested and honed, future CBPR inquiries could greatly benefit from systematically assessing participants’ perception over time and presenting community-level outcomes that are more clearly defined.

## Additional file

Additional file 1: Migrant Health Market Survey. (DOCX 35 kb)

## Abbreviations

CBPR: Community-based participatory research
NHI: National Health Insurance
NIS: New Israeli Sheqel
WTP: Willingness to pay
