# Supplementary material for: Applying a community-based participatory research approach to improve access to healthcare for Eritrean asylum-seekers in Israel: a pilot study
Source: Isr J Health Policy Res. 2017 Nov 15;6:61. doi: 10.1186/s13584-017-0185-9 (PMC5686855; doi:10.1186/s13584-017-0185-9)
Supplement: Additional file 1: — Migrant Health Market Survey. (DOCX 35 kb) [file 13584_2017_185_MOESM1_ESM.docx]

**New - Migrant Health Market Survey**

We are trying to design a way for the Eritrean community to access affordable and comprehensive health insurance equivalent to that of Israeli citizens. This survey will help us understand the exact needs of the community and make sure the community members' voices are heard. Thank you for your cooperation!

Survey Conductor

- Abrahala (T.A.)
- Aklilu (T.A.)
- Awet (Ash)
- Bahta (Jer)
- Baya (T.A.)
- Habte (Ash)
- Hagos (Jer)
- Dewit (Eilat)
- Kifle (T.A.)
- Tsegay (T.A.)
- MigrantHealth (T.A.)
- Nasser (T.A.)
- Yonas (Eilat)

City

- Ashdod
- Eilat
- Jerusalem
- Tel Aviv

1) How old are you?

- 18-24
- 25-34
- 35-44
- 45 and above

2) What year did you arrive in Israel?

- <2005
- 2006
- 2007
- 2008
- 2009
- 2010
- 2011
- 2012
- 2013

3) What is your gender?

- Female
- Male

4) Do you work?

- Yes, I have a full time job
- Yes, I have a full time job and get a tlush
- Yes, I have chikchak jobs
- No
- Other Comments:_______________________________________

5) On average, how much do you earn per month?

- < 2000 NIS
- 2001-4000 NIS
- 4001-6000 NIS
- More than 6001 NIS

6) Do you own a cell phone?

- Yes, I buy scratch cards
- Yes, I pay from my bank account
- No

7) What is your cellular carrier/company?

- Orange
- 012mobile
- Golan
- Pelephone
- Cellcom
- Hot mobile

8) From where do you access the internet?

- Cellphone (data plan)
- Cellphone (WiFi)
- Computer at home
- Computer at Internet café
- Other:__________________________________________________

9) Do you regularly access online social networks?

- Yes, Facebook
- Yes, other
- No

10) Are you worried about losing your job if you have to take time off for being sick?

Select a value from a range of 1, not worried to 5, very worried.

- 1 (Not worried)
- 2
- 3
- 4
- 5 (Very worried)

11) Where do you go when you are sick?

- Arab Health Center in Jerusalem
- Private doctor
- Free clinic in Tel Aviv bus station
- PHR free clinic in Jaffa
- Traditional healer
- Hospital
- Clalit clinic
- Consult religious leader
- I do nothing
- Other:_______________________________________________________________

12) What would be a fair price to pay every month to get the same package of healthcare services available to Israeli citizens for yourself? (Including regular and specialist doctors, drugs from pharmacy, hospital care, etc.)

- <100 NIS per month
- 101-200 NIS
- >300 NIS per month
- Not willing to pay for health insurance

13) Do you have health insurance?

- Yes, through my employer
- Yes, I pay for it privately
- No

14) If you don’t have health insurance, why not?

- I don’t know what health insurance is
- I’m not interested
- I can’t afford it
- The language barrier is too great
- Other:______________________________________________________________

15) If yes, is it clear which services are free under your health insurance and which ones you have to pay for?

Select a value from a range of 1, very clear to 5, not clear at all:

- 1 (Very clear)
- 2
- 3
- 4
- 5 (Not clear at all)

16) If yes, have you ever used your health insurance card to receive medical services?

- Yes
- I tried but the insurance card wasn’t valid
- No
- Other:_______________________________________________________

17) Is it easy for you to make an appointment to see the following doctor?

|  | Very Easy | Somewhat Easy | Not Easy at All |
| --- | --- | --- | --- |
| Free clinic doctor in Tel Aviv central bus station |  |  |  |
| Jerusalem Arab Center doctor |  |  |  |
| Clalit clinic doctor |  |  |  |
| Private doctor |  |  |  |
| PHR doctor free clinic in Jaffa |  |  |  |

18) Do you leave the doctor’s office knowing how to take care of your health better than when you entered the doctor’s office?

Select a value from a range of 1, much better to 5, not better at all

- 1 (Much better)
- 2
- 3
- 4
- 5 (Not better at all)

19) Is it easy for you to communicate your health issue to the doctor or nurse?

Select a value from a range of 1, very easy to 5, very difficult

- 1 (Very easy)
- 2
- 3
- 4
- 5 (Very difficult)

20) How important is it for you to receive the following services? (last column not important at all)

|  | Very important | Somewhat important | |  |
| --- | --- | --- | --- | --- |
| Someone setting up doctor's appointments for you | 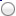 | 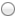 | 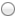 | |
| Someone who will interpret what the doctor says | 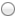 | 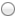 | 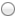 | |
| Someone who will explain to you how to take medicine | 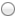 | 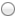 | 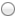 | |
| Someone who will check up on you the following day | 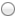 | 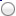 | 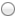 | |
| Understanding your rights related to health | 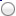 | 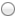 | 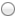 | |
| Learning how to take care of your and your family's health | 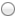 | 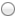 | 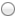 | |

21) What are your main health concerns?

- Stress, constant state of worry, trouble sleeping
- Chronic health conditions (diabetes, high blood pressure, heart disease, etc.)
- Accident or acute illness
- Other:____________________________________________________________

22) If you would like to be contacted to learn more about this service, please write your contact details here:

Name:________________________________________

Phone:_______________________________________

Email:_______________________________________

Comments:

**From here on – relevant only to parents**

23) If you have children younger than 18, are they insured in Meuchedet?

- Yes
- No

24) Are you satisfied with the service at Meuchedet?

- Yes
- No

25) If yes, is it because:

- I understand their system
- The staff and doctors are helpful and patient
- They speak a language I understand
- Other, please specify:

26) If no, is it because:

- I don’t understand their system
- The staff and doctors are not helpful and patient
- They don’t speak a language I understand
- Other, please specify:
